# Service User Involvement in Recovery-Oriented Care Planning: A Realist Synthesis

**DOI:** 10.1192/bjo.2022.202

**Published:** 2022-06-20

**Authors:** Thomas John, Jenny Billings, Patricia Wilson

**Affiliations:** University of Kent, Canterbury, United Kingdom

## Abstract

**Aims:**

Service user involvement (SUI) in recovery-oriented care planning (ROCP) warrants more sophisticated theorisation and explanation to support practice improvement. This study investigated which changes to practice work best, in what circumstances, and to what extent, to embed an active role for service users’ involvement in ROCP during the acute inpatient mental health care pathway.

**Methods:**

A realist synthesis, combined with qualitative methods, was conducted to theoretically explore the causal mechanisms that underlie SUI in ROCP and how contextual factors influence the link between these causal mechanisms and outcomes. The study was conducted in three stages: theory-gleaning, theory-refinement and theory-consolidation. Initial programme theories were developed in the theory-gleaning stage. Theories were refined iteratively in the theory-refinement stage, using evidence from a realist review and interview data. With stakeholder involvement, refined programme theories were finely tuned using ‘if-then’ statements in the consolidation stage.

**Results:**

Five programme theories relating to the acute care pathway were identified following the realist synthesis:

1. **‘Provider-controlled care transition’** (admission to acute inpatient mental health units), referring to limitations to service users’ active involvement. The focus of care and access to acute inpatients units should be needs-led, rather than resource-led or demand-driven;
2. **‘Care plan as a recovery tool?’** – addressing infrastructural and organisational limitations to active SUI in care-plan formulation. The use of multidisciplinary meetings as a forum for care-plan formulation can create a cohesive approach and facilitate shared ownership;
3. ‘**Ward rounds as a non-inclusive arena for shared decision making’**, highlighting their unfulfilled potential for shared decision making about treatment. Professionals should focus on preparing service users for the ward-round process. Opportunities and access for service users to build therapeutic relationships with treating doctors are vital components;
4. **‘Peer support worker intervention'** as a key factor in service users’ recovery’, concerning their positive impact. Their presence in ward rounds and care-planning meetings might create a more user-friendly atmosphere for service users; and
5. **‘Provider-controlled care transition’** (discharge from acute inpatient mental health units), increasing focus on preparing service users for transition into the community, and constraints on resources should not dictate or anticipate decisions on discharging service users.

**Conclusion:**

The study identified practices required to embed an active role for service users to be involved in ROCP, namely multi-contextual interventions at various levels (macro, meso and micro) of the mental health system. The study uncovered barriers that restrain SUI in ROCP, impacting desirable outcomes.

